# Mpox Discourse on Twitter by Sexual Minority Men and Gender-Diverse Individuals: Infodemiological Study Using BERTopic

**DOI:** 10.2196/59193

**Published:** 2024-08-13

**Authors:** Yunwen Wang, Karen O’Connor, Ivan Flores, Carl T Berdahl, Ryan J Urbanowicz, Robin Stevens, José A Bauermeister, Graciela Gonzalez-Hernandez

**Affiliations:** 1 Department of Computational Biomedicine Cedars-Sinai Medical Center West Hollywood, CA United States; 2 William Allen White School of Journalism and Mass Communications University of Kansas Lawrence, KS United States; 3 Department of Biostatistics, Epidemiology and Informatics Perelman School of Medicine University of Pennsylvania Philadelphia, PA United States; 4 Departments of Medicine and Emergency Medicine Cedars-Sinai Medical Center West Hollywood, CA United States; 5 Annenberg School for Communication and Journalism University of Southern California Los Angeles, CA United States; 6 Department of Family and Community Health School of Nursing University of Pennsylvania Philadelphia, PA United States

**Keywords:** mpox, monkeypox, social media, sexual minority, SMMGD, sexual minority men and gender diverse, emerging infectious disease, infectious disease outbreak, health activism, health promotion, health stigma, stigma prevention, health equity, natural language processing, BERTopic

## Abstract

**Background:**

The mpox outbreak resulted in 32,063 cases and 58 deaths in the United States and 95,912 cases worldwide from May 2022 to March 2024 according to the US Centers for Disease Control and Prevention (CDC). Like other disease outbreaks (eg, HIV) with perceived community associations, mpox can create the risk of stigma, exacerbate homophobia, and potentially hinder health care access and social equity. However, the existing literature on mpox has limited representation of the perspective of sexual minority men and gender-diverse (SMMGD) individuals.

**Objective:**

To fill this gap, this study aimed to synthesize themes of discussions among SMMGD individuals and listen to SMMGD voices for identifying problems in current public health communication surrounding mpox to improve inclusivity, equity, and justice.

**Methods:**

We analyzed mpox-related posts (N=8688) posted between October 2020 and September 2022 by 2326 users who self-identified on Twitter/X as SMMGD and were geolocated in the United States. We applied BERTopic (a topic-modeling technique) on the tweets, validated the machine-generated topics through human labeling and annotations, and conducted content analysis of the tweets in each topic. Geographic analysis was performed on the size of the most prominent topic across US states in relation to the University of California, Los Angeles (UCLA) lesbian, gay, and bisexual (LGB) social climate index.

**Results:**

BERTopic identified 11 topics, which annotators labeled as mpox health activism (n=2590, 29.81%), mpox vaccination (n=2242, 25.81%), and adverse events (n=85, 0.98%); sarcasm, jokes, and emotional expressions (n=1220, 14.04%); COVID-19 and mpox (n=636, 7.32%); government or public health response (n=532, 6.12%); mpox symptoms (n=238, 2.74%); case reports (n=192, 2.21%); puns on the naming of the virus (ie, mpox; n=75, 0.86%); media publicity (n=59, 0.68%); and mpox in children (n=58, 0.67%). Spearman rank correlation indicated significant negative correlation (ρ=–0.322, *P*=.03) between the topic size of health activism and the UCLA LGB social climate index at the US state level.

**Conclusions:**

Discussions among SMMGD individuals on mpox encompass both utilitarian (eg, vaccine access, case reports, and mpox symptoms) and emotionally charged (ie, promoting awareness, advocating against homophobia, misinformation/disinformation, and health stigma) themes. Mpox health activism is more prevalent in US states with lower LGB social acceptance, suggesting a resilient communicative pattern among SMMGD individuals in the face of public health oppression. Our method for social listening could facilitate future public health efforts, providing a cost-effective way to capture the perspective of impacted populations. This study illuminates SMMGD engagement with the mpox discourse, underscoring the need for more inclusive public health programming. Findings also highlight the social impact of mpox: health stigma. Our findings could inform interventions to optimize the delivery of informational and tangible health resources leveraging computational mixed-method analyses (eg, BERTopic) and big data.

## Introduction

The ongoing mpox outbreak, which started in May 2022, is the first instance of human-to-human transmission in multiple nonendemic geographical areas [1]. Formerly known as monkeypox and renamed in November 2022 to minimize stigma [2], the current mpox outbreak in humans resulted in 32,063 cases and 58 deaths in the United States and 95,912 cases worldwide by March 5, 2024 [3]. Mpox is an infectious viral disease and a zoonosis: For animal-to-human transmission, a human can contract the virus through coming in contact with or consuming an infected animal from a range of mammalian species or through direct contact with the natural host’s blood or body fluids; for human-to-human transmission, mpox can be spread via direct skin-to-skin contact [4]. Since the first identification of mpox virus among laboratory monkeys in 1957 and the first report of mpox in humans in 1970, mpox has been largely confined to endemic areas in Africa, except for a small 2003 outbreak in the United States, where transmissions occurred from infected animals to dozens of humans [1]. Common symptoms of mpox in humans include rash, lymphadenopathy, fever, enlarged lymph nodes, headache, chills/rigors, fatigue, dysphagia/swallowing difficulty, nausea/vomiting, and conjunctivitis [5].

Although public health surveillance research on the human mpox outbreak has evaluated a contact-tracing information system [6] and surveyed sexual and gender minorities for their vaccination intention against mpox [7], the health communication aspect and the implication of current public health approaches to mpox on stigmatization and discrimination have not been sufficiently addressed. During disease outbreaks, stigmatizing and discriminating against certain at-risk groups challenge public health efforts. This occurred during the COVID-19 pandemic in the United States, where hate crimes and violence toward people of East Asian descent surged due to perceptions attributing the pandemic to China [8]. Similarly, sexual minority men and gender-diverse (SMMGD) individuals have been battling with the so-called “gay disease” stigma attached to HIV/AIDS since the 1980s, when the infection and death cases were initially reported in North America to be prevalent among SMMGD individuals [9]. The recent mpox outbreak, coupled with rising misinformation [10], stigma [11,12], and conspiracy theories [13], may, like HIV, experience a stigmatization process that leads to delayed care seeking and further marginalization of SMMGD individuals.

In the recent literature on health stigma and equity, researchers argue that the special focus of public health agencies and media on SMMGD individuals during the mpox epidemic may fuel stigma and homophobia but not help clinically [11,14]. However, the current scientific literature about mpox poorly represents the voices of this community. The literature either features expert opinions not based on original empirical research [11] or analyzes public opinion generally [14]. Few studies have directly examined insights from SMMGD individuals, who are arguably impacted the most by mpox epidemiologically and infodemiologically. Even in a study that did focus on mpox and this community, the analyses were performed on mpox tweets containing keywords about lesbian, gay, bisexual, transgender, queer/questioning, intersex, and other sexual or gender identities (LGBTQI+), and the posts were from general users not necessarily self-identified as LGBTQI+ [12]. Not specific to mpox, 1 study [15] found a more negative patient experience sentiment among LGBTQI+ users than non-LGBTQI+ users, which demonstrates the importance of considering the post author’s identity when analyzing social media data. This study addresses this research gap by analyzing online mpox posts from users who self-identify as SMMGD specifically and by discussing the implications of our findings for health communication tackling mpox and future disease outbreaks, with an emphasis on fairness, equity, and stigma prevention and control.

Methodologically, this study applied natural language processing (NLP) methods in a health context [16]. We investigated the best practices when applying BERTopic [17], a topic-modeling technique based on the language model Bidirectional Encoder Representation from Transformers (BERT) [18]. Topic modeling is a widely used text classification method for identifying topics in a collection of documents, such as social media posts, using approaches such as latent Dirichlet allocation (LDA) [19]. BERTopic [17] is a more recent topic-modeling technique that has gained popularity for its ease of interpretation and ability to leverage Hugging Face transformers and class-based Term Frequency–Inverse Document Frequency (c-TF-IDF) to create dense clusters. According to a study comparing the efficacy of 4 popular topic-modeling approaches, namely LDA [19], nonnegative matrix factorization (NMF) [20], Top2Vec [21], and BERTopic [17] on a Twitter data set, BERTopic was found to perform exceptionally well and able to, like NMF, provide a clearer distinction between identified topics than LDA and Top2Vec; compared to NMF, BERTopic provides more novel insights using its embedding approach [22]. We also investigated how to best validate and construe multiclass machine classification results through follow-up analyses.

## Methods

### Data Collection and Descriptions

The sample of this study consisted of mpox-related tweets in English (N=8688) posted between October 10, 2020, and September 20, 2022, by 2326 users who self-identified on Twitter/X as SMMGD individuals. They belonged to a cohort of 10,043 users whom a previous study [23] predicted as users who self-identify as gay, bisexual, or men who have sex with men based on tweets and profile descriptions, with a reported accuracy rate of 85%.

For this study, we used the official Twitter application programming interface (API) and an in-house Python script to collect the tweet timelines of the users identified in the prior study [23]. We filtered the timeline data using mpox-related keywords (ie, “monkeypox,” “hmpxv,” “monkey pox,” and “mpox”), yielding a subset of tweets from 2687 users who discussed mpox.

Of the 2687 users, we retained 2326 (86.56%) users who self-reported as SMMGD. Two annotators verified each user’s gender and sexual profile based on their profile descriptions and historical timelines. The user validation process is described next.

### Validation of Gender/Sexual Identity Self-Reports

The validation was at the user level. First, we curated an evidence data set not specific to mpox for the 2687 users, including their profile descriptions or tweets containing SMMGD gender/sexual identity keywords (see Multimedia Appendix 1). Second, informed by the evidence data set, we developed annotation guidelines (available upon request), which were then discussed, refined, and agreed upon among the research team. Third, 2 annotators double-coded 20% (n=537) of users with their profile descriptions and tweets matching SMMGD sexual/gender identity keywords, and the annotators reached substantial intercoder reliability (Cohen κ=0.763) [24]. Lastly, the 2 annotators independently validated the remaining 80% (n=2150) of the users.

Through validation, we were left with 2326 SMMGD individuals and their 8688 tweets about mpox, which was the final data set used for analysis.

### Ethical Considerations

All data used in this study were collected in accordance with Twitter/X terms of use and were publicly available at the time of collection. The Institutional Review Board of the University of Pennsylvania reviewed the protocol regarding gender/sexual identity investigation and deemed it exempt from review under Category (4) of Paragraph (b) of the US Code of Federal Regulations Title 45 Section 46.101 for publicly available data sources (45 CFR §46.101(b)(4)).

The research team, including the annotators, included members from the LGBTQI+ community who were familiar with LGBTQI+ terms and language use, and the research team diversely included clinicians, bioinformaticians, data scientists, and social scientists. These were key to ethical handling, analysis, and interpretation of the study data. We also made cautious efforts in conducting and reporting this research, including deidentifying data to the largest extent, providing password-protected access to only known study personnel, and not open-sourcing the data set in publicly available repositories.

All example posts quoted in this study were modified or paraphrased without changing the meaning in order to reduce searchability and protect user anonymity.

### Data Preprocessing

To prepare the data for BERTopic modeling, we cleaned and normalized the text in the following steps: (1) expanding contractions (eg, from “I’m” to “I am”); (2) translating emojis and emoticons to text; (3) removing HTTP/HTTPS links, the hashtag sign (ie, #), user mentions (ie, @user_name), special characters, and extra spaces; (4) using lowercase; and (5) converting a list of context-specific words of multiple forms to 1 standard form using a self-defined dictionary. For example, “monkeypox,” “mpx,” “hmpxv,” and other forms of reference to mpox were converted to the standard form “mpox.” This prevented high-frequency context-specific synonyms from occurring separately in topic representations, hence enhancing model performance by increasing the topic word diversity. See Multimedia Appendix 2 for source words and normalized words.

Next, we applied the *nltk* Python package to tokenize the text, remove stop words, and perform lemmatization.

### Implementation of BERTopic Modeling

The preprocessed posts were then passed to the BERTopic model following 6 steps: (1) transforming documents into numerical representations using *all-MiniLM-L6-v2*, a sentence transformer model capable of capturing semantic similarity between documents; (2) using Uniform Manifold Approximation and Projection (UMAP) [25] to reduce the dimensionality of input embeddings, preparing them for topic clustering; (3) applying Hierarchical Density-Based Spatial Clustering of Applications with Noise (HDBSCAN) [26], a hierarchical clustering algorithm, to find the natural groupings or topic structure of the documents; (4) implementing *sklearn*’s CountVectorizer to convert the collection of documents to a matrix of token counts, namely a bag-of-words representation; (5) adding a c-TF-IDF representation to the BERTopic model, with additional BM-25 weighting to reduce frequent words in topic generation; and (6) fine-tuning the BERTopic representation by adjusting hyperparameters, including *n_neighbors*, *n_components*, *min_dist*, *min_cluster_size*, and *min_samples*.

### Qualitative Synthesizing and Validation of Machine-Generated Topic Results

Given the machine-generated topics, 2 annotators judged the top 10 keywords and the most representative tweets of each topic to create semantic topic labels. This resulted in a k-topic label scheme. The research team read more tweets and refined the topic labels to make them more summative, preventing the labels from overfitting the example tweets reviewed.

To evaluate the quality of topic representation and the generalizability of the k-topic label scheme to unseen instances, a primary annotator read 10% (n=864) of the sample and categorized the tweets to topics without seeing the machine-assigned labels, and a secondary annotator read 15% (n=130) of this subset to test interrater reliability. The topic assignments of the human annotators were individually compared to those of the machine to evaluate the BERTopic model performance. The percentage agreement rates between the machine and the primary human annotator, between the machine and the secondary human annotator, and between the 2 human annotators were 60.1%, 60%, and 70.77%, respectively.

### Testing Geographic Association With Topic Sizes

The size of the topics was compared across the US states (geolocations were extracted from location information in tweet metadata and user profiles using the Carmen 2.0 tool) [27]. The size of the topics indicates their relative salience, and the measure was standardized based on the total number of posts in a state before being used in geographic analyses.

To test the geolocation-based associations between online mpox discussion patterns and local social variables at the US state level, we adopted the lesbian, gay, and bisexual (LGB) social climate index aggregated by the Williams Institute at the University of California, Los Angeles (UCLA) [28] to indicate the level of social acceptance of LGB individuals of each US state. We asked the following research question: Is health activism, the most prominent topic in this corpus, more prevalent in states with a higher or a lower LGB social climate index? A positive correlation would suggest that health activism is more active when the social climate is more supportive, whereas a negative correlation would suggest greater health activism in the face of oppression.

## Results

### Topic Representations

The BERTopic procedure identified 11 topics in this data set (N=8688) and assigned 1 topic representation per tweet. We leveraged both machine-generated results and human interpretation to generate topic labels.

**Figure 1:**
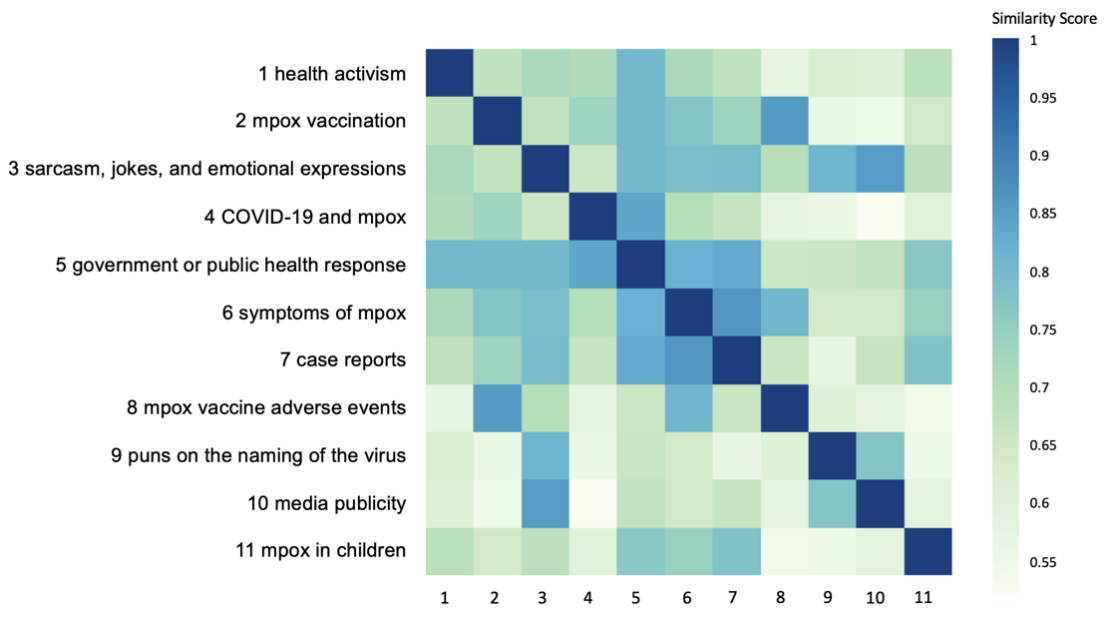
Topic similarity matrix.

Figure 1 shows the semantic similarity between topics. Table 1 provides the size, label, top 10 keywords, and example posts (paraphrased) of each topic. Ranked by topic size, the topics and their definitions were as follows:

- Health activism (n=2590, 29.81%)
- Mpox vaccination (n=2242, 25.81%)
- Sarcasm, jokes, and emotional expressions (n=1220, 14.04%)
- COVID-19 and mpox (n=636, 7.32%)
- Government or public health response (n=532, 6.12%)
- Symptoms of mpox (n=238, 2.74%)
- Case reports (n=192, 2.21%)
- Mpox vaccine adverse events (n=85, 0.98%)
- Puns on the naming of the virus (n=75, 0.86%)
- Media publicity (n=59, 0.68%)
- Mpox in children (n=58, 0.67%)

**Table 1:** The 11 topics found by the BERTopic^a^ algorithm and labeled by human annotators, with the topic size, top keywords, and example posts (modified or paraphrased without changing the meaning to reduce searchability and protect anonymity) of each topic.

Topic number; posts, n (%)	Label	Top 10 keywords	Example posts
Topic 1; 2590 (29.81)	Health activism	“gay,” “men,” “sex,” “sti,” “community,” “spread,” “contact,” “hiv,” “disease,” “sexual”	Dear straight people, mpox is not an STD^b^, and it doesn’t only affect gay men… Read this entire thread [URL]. MONKEYPOX IS NOT A GAY DISEASE. @user Anyone can get monkeypox. Anyone can transmit. You don’t have to have sex to transmit it. @user This is related to gay history in the U.S. that is not being covered as part of public education. Anyone who knows anything about how HIV/AIDS was handled in the U.S. immediately sees the bullshit going on with mpox, but to most (straight) people, this whole situation is brand new. I don’t know what the future holds. At a time when we have militias harassing us and elected officials musing about bringing sodomy laws back, the WHO^c^ saying that Mpox primarily affects LGBTQ^d^ people is giving a loaded handgun to a group with an itchy trigger finger.
Topic 2; 2242 (25.81)	Mpox vaccination	“vaccine,” “vaccination,” “appointment,” “got,” “smallpox,” “available,” “vaccinated,” “dos,” “dose,” “line”	Any updates on monkeypox vaccine availability? Out here getting the monkeypox vaccine, keeping our community safe I had to get my monkeypox vaccination in Canada as my country [US] has failed horribly on the vaccine rollout. If you are in SF or know people in SF, please retweet. @user has a great system in place to get your monkeypox vaccine. Monday-Friday 8am-Noon. BREAKING news: Moderna is reportedly beginning research and testing for a monkeypox vaccine. DeSantis has already refused to declare a public health emergency in Florida for mpox, though that would speed resources, while Florida is in dire need of mpox vaccines and treatments… Africa, Continent with Mpox Deaths, Has No Vaccine [URL].
Topic 3; 1220 (14.04)	Sarcasm, jokes, and emotional expressions	“face,” “joy,” “tear,” “shit,” “really,” “going,” “lol,” “want,” “heart,” “know”	Thank you for an even-handed sober article on #monkeypox with wise words from @user via ** News. Mpox is a nightmare. There could be a far worse scenario ahead. So many aspects of mpox are exhausting and infuriating far BEYOND just being concerned about how to stay as safe and protected as possible, but… Yeah, ok “a wait-and-see response” is sadly far too common in modern healthcare.
Topic 4; 636 (7.32)	COVID-19 and mpox	“covid,” “pandemic,” “polio,” “going,” “mask,” “climate,” “like,” “u,” “just,” “virus”	If we do not take national safety measures for monkeypox, then we really have learned nothing from COVID. Reading about monkeypox outbreaks while quarantining with COVID. [URL]
Topic 5; 532 (6.12)	Government or public health response	“emergency,” “outbreak,” “biden,” “health,” “public,” “response,” “cdc,” “declares,” “administration,” “state”	Two months into the monkeypox outbreak that has spiraled into exponential spread, the White House has finally announced that it will eventually appoint a point person for it. [URL]
Topic 6; 238 (2.74)	Symptoms of mpox	“symptom,” “lesion,” “test,” “painful,” “diagnosis,” “tested,” “doctor,” “face,” “body,” “sore”	I don’t like the disconnected mpox cases around the world. This becomes more difficult to trace. Doctors seeing patients must pay close attention to travel history and these distinct skin lesions on face, hands, and feet. Eventually there’re no treatments for mpox. So, for those who have exposure and are symptomatic, they cannot find testing. They should isolate until symptoms resolve and lesions heal. The CDC^e^ should also monitor the presumptive positive cases based on known exposure symptoms. How long does it take for mpox symptoms to resolve? If it is just a few days, then yes, people should stay home from work. If it takes a few weeks for symptoms and sores to resolve, then sure, people just need to cover them up and go to work.
Topic 7; 192 (2.21)	Case reports	“case,” “confirmed,” “test,” “testing,” “county,” “spain,” “reported,” “provider,” “number,” “tested”	From the New York State Department of Public Health #mpox [URL] What about the man who had traveled from Mexico and died in Texas several weeks ago from monkeypox?... I still maintain that we’ve had even more than that. They’ve just not been listed after purposefully not being tested.
Topic 8; 85 (0.98)	Mpox vaccine adverse events	“arm,” “injection,” “shot,” “lump,” “site,” “red,” “bump,” “swelling,” “itch,” “pox”	The lump in my left arm from the monkeypox vaccine continues to grow. I’m beginning to fear. [Image]
Topic 9; 75 (0.86)	Puns on the naming of the virus	“monkey,” “evil,” “banana,” “pizza,” “gia,” “shawn,” “blocked,” “star,” “red,” “speak”	No more lies mpox, see no evil monkey, hear no evil monkey, speak no evil monkey. Got my monkeypox vaccine… Having Fried Banana for dinner, Banana Pudding as the side dish, and Bananas Foster for dessert. Also, never realized how much easier it is to peel a banana with my feet.
Topic 10; 59 (0.68)	Media publicity	“oliver,” “john,” “beto,” “hbo,” “tonight,” “magnet,” “sean,” “rourke,” “oz,” “hannity”	Big shoutout to John Oliver for addressing the homophobia around Mpox #Mpox: Last Week Tonight with @John Oliver [URL] via @YouTube
Topic 11; 58 (0.67)	Mpox in Children	“child,” “school,” “kid,” “rare,” “chain,” “daycare,” “case,” “report,” “transmission,” “younger”	According to a vast amount of global data, mpox cases remain very rare in children in the US and elsewhere. No evidence of transmission in schools or of substantial transmission chains among children peer groups. This is unscientific fear mongering about the virus as there have been no sustained transmission chains of mpox among children reported this week.

^a^BERTopic was able to classify 91.24% (n=7927) of the data, with the remaining 8.76% (n=761) tweets labeled as outliers, or noise. The noise data could be forcefully classified into 1 of the 11 topics, but it might not substantially aid our understanding of this corpus.

^b^STD: sexually transmitted disease.

^c^WHO: World Health Organization.

^d^LGBTQ: lesbian, gay, bisexual, transgender, queer/questioning.

^e^CDC: Centers for Disease Control and Prevention.

#### Health Activism

Health activism refers to efforts promoting equity, fairness, and justice on a health agenda; it is aimed to counter challenges in the existing power dynamics perceived to negatively impact health communication or health outcomes [29]. In this corpus, most of the tweets center around health activism addressing awareness, homophobia, and the health stigma related to mpox.

Some posts on this topic educate the public about the mpox transmission mechanism, stressing that mpox is not a sexually transmitted infection/sexually transmitted disease (STI/STD) and not a “gay disease.” Specifically, the epidemiological focus on the gay community is perceived by the users to have amplified an association between the gay community and mpox:

@user_name The epidemiological criteria has created impediments to testing people outside the gay community, which has ensured that the gay community remains the most visible in criteria and counts, despite the fact that mpox is transmissible through various kinds of contact.

Like this example, these posts sometimes engage with other users through the *@mention* function of Twitter/X to spark conversations.

This group of posts also references the HIV endemic in discussing mpox. Some note the similar discursive pattern between mpox and HIV, as they both tend to stigmatize the queer community. Some discuss the policy implications of existing problematic speech surrounding mpox, warning against discriminative public policies targeting SMMGD individuals.

#### Mpox Vaccination

The second-largest group of posts discuss various issues about the mpox vaccine. These posts include the importance of vaccination against mpox, inquiries or exchange of information for getting the vaccine, announcements of vaccine appointments or having received vaccination, and other news on the mpox vaccine.

Notably, geographical disparity regarding health resources is a recurring subtheme in this topic, with specific geolocations, such as New York City, mentioned in discussing national or international vaccine shortage.

#### Sarcasm, Jokes, and Emotional Expressions

The third-largest group of posts focuses on rhetoric and emotional expressions related to mpox, including thankfulness, fear, sadness or anger, and sarcasm or jokes.

#### COVID-19 and Mpox

With the lingering effects of COVID-19 on public awareness, this fourth-largest topic is characterized by discussing mpox in comparison, or in relation, to COVID-19 in public health measures, resources, etc.

#### Government or Public Health Response

This group of posts starts with the state or federal government declaring mpox as a public health emergency. As the outbreak developed, more tweets were posted discussing various public health responses to the mpox outbreak, where we observed critiques on the administration.

#### Symptoms of Mpox

This topic contains posts delineating specific symptoms (eg, skin lesions on the face, hands, and feet), sometimes accompanied by images, and posts mentioning mpox disease symptoms as an aspect of this issue (eg, how long it takes symptoms to resolve and the implications of symptom features for work and community transmission).

#### Case Reports

In this topic, most tweets are about case numbers at the national, state, or city level, and there is individual case reporting as well.

#### Mpox Vaccine Adverse Events

About 1% (n=85) of the data are distinguished by the machine from the second-largest topic, “mpox vaccination,” to specifically discuss the adverse events related to the mpox vaccine. The adverse events include lumps, bumps, swelling, and itchiness in the arm where the injection is received.

#### Puns on the Naming of the Virus

Despite being renamed as mpox, the term “monkeypox” is still referenced frequently by laypersons online. This group of posts includes puns on the previous naming of the virus around “monkey.”

#### Media Publicity

Some posts either praise or criticize or mention in a neutral tone such popular media as television shows for their discussions about mpox. For example, a subgroup of posts praised *Last Week Tonight with John Oliver* for addressing lesbian, gay, bisexual, transgender, queer/questioning, asexual (LGBTQA) self-care, as well as homophobia related to mpox; other posts mentioned Fox’s *The Dr. Oz Show* for its explanation on the theory of mpox’s origin.

#### Mpox in Children

The last topic centers around whether and how mpox affects children. Posts mostly state that mpox is rare in children and that no evidence suggests a transmission chain in children’s peer group. Occasionally, there are also messages about antifearmongering or debunking of misinformation/disinformation, such as queer teachers tend to spread mpox to school kids. Despite constituting the smallest group in this corpus, these posts focus on a specific vulnerable population and are worthy of close attention.

### Geographic Associations Between Health Activism and the LGB Social Climate

**Figure 2:**
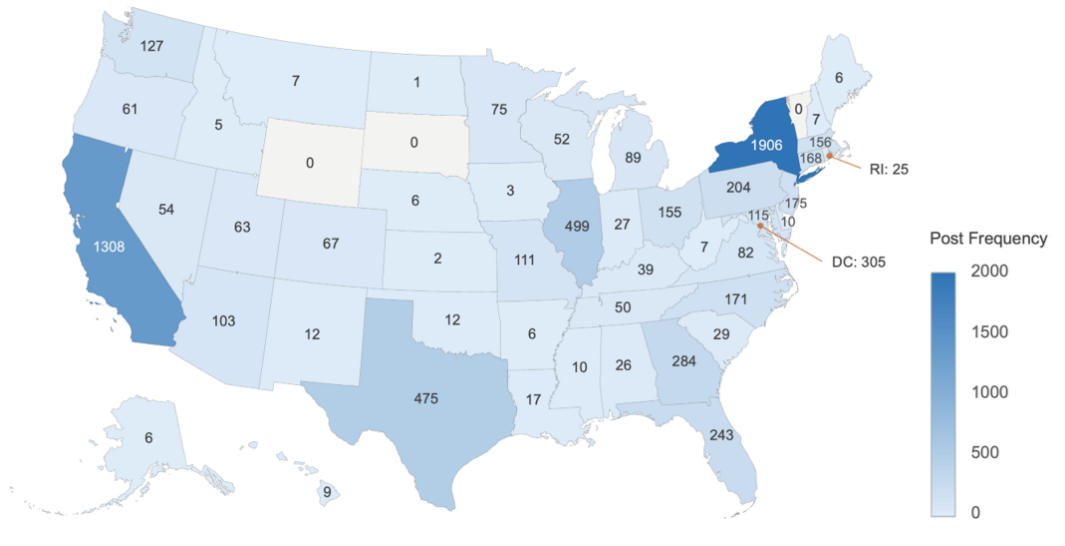
Geographic heatmap of post frequency at the US Census state level. The state of New York and the state of California had the most posts on mpox in the study data set.

**Figure 3:**
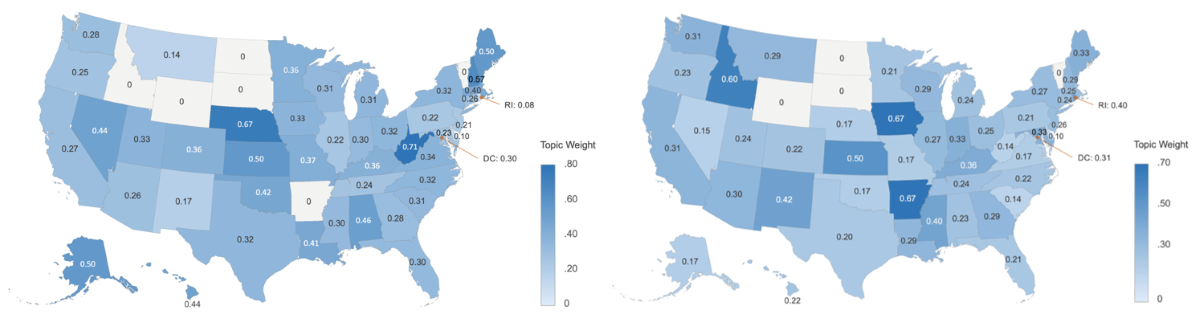
Topic 1 (health activism) and topic 2 (mpox vaccination) weights per US Census state. Calculated as the number of topic 1 or 2 posts posted in a US state divided by the total number of posts from that state, the topic weight measures the relative importance of a certain topic in a US state.

The Figure 2 heatmap visualizes the state-level raw frequencies of posts. Figure 3 heatmaps geographically show the standardized topic sizes or within-state topic weights (ie, the proportion of posts assigned to a topic out of all posts from that state, as explained in the *Methods* section) of topic 1, “health activism,” addressing awareness, homophobia, and the health stigma related to mpox, and topic 2, “mpox vaccination,” discussing appointments, vaccination status, and geographical disparity in vaccine resources.

A Spearman rank correlation test was performed between the state-level standardized topic size of health activism and the state-level LGB social climate index. Results indicated a negative correlation (ρ=–0.322, *P*=.03), suggesting that health activism about mpox is more active in US states with less LGB social acceptance.

## Discussion

### Principal Findings

The public response to the mpox outbreak highlights a long-standing problem: disease outbreaks often trigger health stigma, and history repeats itself by further marginalizing the at-risk communities perceived to be linked to certain diseases. This study combined computational and human-directed strategies to closely examine mpox-related online discussions among SMMGD individuals on Twitter/X. Our findings fill the gap in current research, which is not explicitly inclusive of LGBTQI+ perspectives, as it does not focus on the discourse among SMMGD users. Based on the 11 topics we identified, engaged argumentation is, indeed, captured in our collection, with multifaceted mpox discussions including health activism (29.81%); mpox vaccination (25.81%); sarcasm, jokes, and emotional expressions (14.04%); COVID-19 and mpox (7.32%); government or public health response (6.12%); symptoms of mpox (2.74%); case reports (2.21%); mpox vaccine adverse events (0.98%); puns on the naming of the virus (0.86%); media publicity (0.68%); and mpox in children (0.67%). Although utilitarian content (eg, vaccine access, case reports, and mpox symptoms) is part of this corpus, other types of content also address the relation of mpox to social justice and equity—those messages focus on increasing awareness and advocating against homophobia and the health stigma related to mpox.

Compared to previous research that does not focus on SMMGD voices, our study found that SMMGD individuals strongly oppose the narratives that “mpox is an STI/STD” and “mpox is a gay disease,” as the spread of such misinformation/disinformation can prevent the public from understanding the transmission mechanisms of mpox. It also risks exacerbating homophobia and health stigma by associating a disease with the community. Thus, in addition to highlighting social media users’ disclosure of attention on mpox prevention and control, our study also sheds light on the social implications of disease outbreaks, suggesting that the public health field should consider how public discourse around infectious diseases could contribute to biases and inequities in public health.

In line with prior research that found links between HIV and homophobia [9] and between COVID-19 and anti-Asian racism [8], this study found that mpox is linked to homophobia. In addition, our geographic analyses suggest that health activism is more likely to be motivated by an oppressive (rather than accepting) social climate in the United States, suggesting a resilient communicative pattern among SMMGD individuals in the face of public health oppression. This finding requires further investigation, given our somewhat limited collection from some geographic locations.

Regarding other public health implications, our findings suggest areas where community needs are not met and where public health sectors can improve. For example, in the second-largest topic found by this study (ie, mpox vaccination), geographical disparity regarding health resources is a recurring subtheme: SMMGD individuals pointed out vaccine shortages both in the United States (eg, New York City) and other areas (eg, Africa). Garnering real-time feedback directly from a target population provides a way to evaluate public health programming (eg, regarding mpox vaccine rollouts). The findings of this study also reveal what questions are being asked about mpox by the community affected by it, which could help inform public health interventions, such as social media campaigns or strategies for distributing tangible and informational resources.

Throughout this study, we used BERTopic modeling as a cost-effective method of synthesizing community and patient perspectives from big data that, especially when implemented in a timely fashion, could directly benefit public health practices. Users’ affect-enriched praises and criticisms of the government administration and media regarding their response to the human mpox outbreak also evidence how social media enables political/media engagement for social change. From the perspectives of nonprofit health organizations and government agencies, future public health efforts could use real-time monitoring of social media content to support health activism and strategically plan messages to combat and correct misinformation during disease outbreaks, a time of crisis that is prone to fear mongering and the propagation of inaccurate, false, or misleading health-related messages that can erode both health promotion efforts and clinical efforts.

### Strengths

Methodologically, this study contributes to the current literature in 3 aspects. First, we showed how to leverage a previously published data set of perspectives from a cohort of our interest to derive and validate a subset of data for another use case. Second, we detailed how to apply BERTopic for categorizing the themes arising from big data, amplifying marginalized voices by synthesizing unstructured text data into a structured form for interpretation and knowledge extraction. Third, we validated BERTopic results as a multiclass classification task via 3-way comparisons: (1) human annotator 1 versus human annotator 2, (2) machine versus human annotator 1, and (3) machine versus human annotator 2. This validation strategy considers the inherent difficulty of multiclass classification in machine learning, and it assesses the performance of the machine model after adjusting for how well human annotators achieve interrater reliability.

### Limitations

We validated machine-generated topic results on 10% of the data instead of all posts, but this is in line with standard validation practices [30] in biomedical informatics and considered sufficient to assess the validity of the automatic large-scale analysis of the entire data set. Some posts may belong to more than 1 topic, but BERTopic assigns the most prominent topic to each post. In future research, we will explore how to analyze data when multiple topics are assigned to each post. This study was based on 8688 publicly available posts from 2326 SMMGD individuals, who were identified through a previous study of our team [23]. Although the cohort represented users across different US states [23], the generalizability of our findings to the broader population may still be limited to the level of representativeness of the cohort, a limitation that has been discussed in previous health research involving social media data as well [31].

### Conclusion

In conclusion, this study highlights the response of a large group of SMMGD individuals to the mpox outbreak. BERTopic results revealed 11 topics around 2 themes: (1) affective and political expressions, such as health activism against mpox misinformation/disinformation and stigma, and (2) utilitarian information exchange on the mpox vaccine and its adverse effects, mpox symptoms, case reports, and public health measures. Acknowledging and including these perspectives in public health programming is key to enhancing equity, inclusion, and fairness/justice, while optimizing health resource planning and allocation.

Through this study, we gathered a previously neglected segment of public opinions and performed “social listening” [32] by analyzing a large social media data set using NLP and machine learning methods. The results of this study could inform clinical practice and health research, improving communication about mpox and future infectious disease outbreaks. For future communication on disease outbreaks to be engaging and effective, clinicians and researchers must efficiently include the perspective of the target population impacted by the disease.

Ultimately, this study calls attention to the social implications of infectious disease outbreaks, including (1) the mechanism by which disease outbreaks tend to trigger hate, prejudice, and stigma attached to at-risk groups (and how stigma prevention and control should underlie public health surveillance and practices) and (2) the need for continuously identifying and addressing gaps in public health programming, such as in the delivery of informational and tangible health resources through social listening to nonexpert community perspectives.

## Appendix

Multimedia Appendix 1 Regular expressions used by Klein et al [23] to detect sexual/gender identities in Twitter/X profiles and tweets.

Multimedia Appendix 2 Word normalization rules.

## Abbreviations

CDC: Centers for Disease Control and Prevention
c-TF-IDF: class-based Term Frequency–Inverse Document Frequency
LDA: latent Dirichlet allocation
LGB: lesbian, gay, and bisexual
LGBTQI+: lesbian, gay, bisexual, transgender, queer/questioning, intersex, and other sexual or gender identities
NLP: natural language processing
NMF: nonnegative matrix factorization
SMMGD: sexual minority men and gender diverse
STD: sexually transmitted disease
STI: sexually transmitted infection
UCLA: University of California, Los Angeles

## Data Availability

Due to the evolving Twitter/X data policies and to protect users, the data set used in this study is not open-sourced. However, access to deidentified tweets for noncommercial research purposes is available on a case-by-case basis upon request to the corresponding author, GGH. The code for this study is also available from the corresponding author upon reasonable request. In addition, the annotation guidelines document is available upon request.
