# Supplementary material for: Mpox Discourse on Twitter by Sexual Minority Men and Gender-Diverse Individuals: Infodemiological Study Using BERTopic
Source: JMIR Public Health Surveill. 2024 Aug 13;10:e59193. doi: 10.2196/59193 (PMC11350314; doi:10.2196/59193)
Supplement: Multimedia Appendix 2 [file publichealth_v10i1e59193_app2.docx]

**Word normalization rules**

(Source Words: Normalized Word)

monkeypox, monkey pox, mpox, mpx, hmpxv: mpox

antivaxxer, antivaxxers: antivaxxer

vaccinated, vaxxed, vaxed: vaccinated

vaccine, vaccines, vax, vaxx, vaxs: vaccine

outbreak, out break: outbreak

sti, stis: sti

std, stds: std

covid, covid 19, covid19: covid
